# Work stress, mental health, and employee performance

**DOI:** 10.3389/fpsyg.2022.1006580

**Published:** 2022-11-08

**Authors:** Biao Chen, Lu Wang, Biao Li, Weixing Liu

**Affiliations:** ^1^School of Business, Zhengzhou University, Zhengzhou, China; ^2^Henan Research Platform Service Center, Zhengzhou, China

**Keywords:** COVID-19, work stress, mental health, employee performance, social uncertainty

## Abstract

The COVID-19 pandemic outbreak—as a typical emergency event—significantly has impacted employees' psychological status and thus has negatively affected their performance. Hence, along with focusing on the mechanisms and solutions to alleviate the impact of work stress on employee performance, we also examine the relationship between work stress, mental health, and employee performance. Furthermore, we analyzed the moderating role of servant leadership in the relationship between work stress and mental health, but the result was not significant. The results contribute to providing practical guidance for enterprises to improve employee performance in the context of major emergencies.

## Introduction

Small- and medium-sized enterprises (SMEs) are the key drivers of economic development as they contribute >50, 60, 70, 80, and 90% of tax revenue, GDP, technological innovation, labor employment, and the number of enterprises, respectively. However, owing to the disadvantages of small-scale and insufficient resources (Cai et al., 2017; Flynn, 2017), these enterprises are more vulnerable to being influenced by emergency events. The COVID-19 pandemic outbreak—as a typical emergency event—has negatively affected survival and growth of SMEs (Eggers, 2020). Some SMEs have faced a relatively higher risk of salary reduction, layoffs, or corporate bankruptcy (Adam and Alarifi, 2021). Consequently, it has made employees in the SMEs face the following stressors during the COVID-19 pandemic: First, employees' income, promotion, and career development opportunities have declined (Shimazu et al., 2020). Second, as most employees had to work from home, family conflicts have increased and family satisfaction has decreased (Green et al., 2020; Xu et al., 2020). Finally, as work tasks and positions have changed, the new work environment has made employees less engaged and less fulfilled at work (Olugbade and Karatepe, 2019; Chen and Fellenz, 2020).

For SMEs, employees are their core assets and are crucial to their survival and growth (Shan et al., 2022). Employee work stress may precipitate burnout (Choi et al., 2019; Barello et al., 2020), which manifests as fatigue and frustration (Mansour and Tremblay, 2018), and is associated with various negative reactions, including job dissatisfaction, low organizational commitment, and a high propensity to resign (Lu and Gursoy, 2016; Uchmanowicz et al., 2020). Ultimately, it negatively impacts employee performance (Prasad and Vaidya, 2020). The problem of employee work stress has become an important topic for researchers and practitioners alike. In this regard, it is timely to explore the impact of work stress on SME problems of survival and growth during emergency events like the COVID-19 pandemic.

Although recent studies have demonstrated the relationship between work stress and employee performance, some insufficiencies persist, which must be resolved. Research on how work stress affects employee performance has remained fragmented and limited. First, the research into how work stress affects employee performance is still insufficient. Some researchers have explored the effects of work stress on employee performance during COVID-19 (Saleem et al., 2021; Tu et al., 2021). However, they have not explained the intermediate path, which limits our understanding of effects of work stress. As work stress causes psychological pain to employees, in response, they exhibit lower performance levels (Song et al., 2020; Yu et al., 2022). Thus, employees' mental health becomes an important path to explain the relationship mechanism between work stress and employee performance, which is revealed in this study using a stress–psychological state–performance framework. Second, resolving the mental health problems caused by work stress has become a key issue for SMEs during the COVID-19 pandemic. As the core of the enterprise (Ahn et al., 2018), the behavior of leaders significantly influences employees. Especially for SMEs, intensive interactive communication transpires between the leader and employees (Li et al., 2019; Tiedtke et al., 2020). Servant leadership, as a typical leader's behavior, is considered an important determinant of employee mental health (Haslam et al., 2020). Hence, to improve employees' mental health, we introduce servant leadership as a moderating variable and explore its contingency effect on relieving work stress and mental health.

This study predominantly tries to answer the question of how work stress influences employee performance and explores the mediating impact of mental health and the moderating impact of servant leadership in this relationship. Mainly, this study contributes to the existing literature in the following three ways: First, this research analyzes the influence of work stress on employee performance in SMEs during the COVID-19 pandemic, which complements previous studies and theories related to work stress. Second, this study regards mental health as a psychological state and examines its mediating impact on the relationship between work stress and employee performance, which complements the research path on how work stress affects employee performance. Third, we explore the moderating impact of servant leadership, which has been ignored in previous research, thus extending the understanding of the relationship between the work stress and mental health of employees in SMEs.

To accomplish the aforementioned tasks, the remainder of this article is structured as follows: First, based on the literature review, we propose our hypotheses. Thereafter, we present our research method, including the processes of data collection, sample characteristics, measurement of variables, and sample validity. Subsequently, we provide the data analysis and report the results. Finally, we discuss the results and present the study limitations.

## Theoretical background and hypotheses

### Work stress and employee performance

From a psychological perspective, work stress influences employees' psychological states, which, in turn, affects their effort levels at work (Lu, 1997; Richardson and Rothstein, 2008; Lai et al., 2022). Employee performance is the result of the individual's efforts at work (Robbins, 2005) and thus is significantly impacted by work stress. However, previous research has provided no consistent conclusion regarding the relationship between work stress and employee performance. One view is that a significant positive relationship exists between work stress and employee performance (Ismail et al., 2015; Soomro et al., 2019), suggesting that stress is a motivational force that encourages employees to work hard and improve work efficiency. Another view is that work stress negatively impacts employee performance (Yunus et al., 2018; Nawaz Kalyar et al., 2019; Purnomo et al., 2021), suggesting that employees need to spend time and energy to cope with stress, which increases their burden and decreases their work efficiency. A third view is that the impact of work stress on employee performance is non-linear and may exhibit an inverted U-shaped relationship (McClenahan et al., 2007; Hamidi and Eivazi, 2010); reportedly, when work stress is relatively low or high, employee performance is low. Hence, if work stress reaches a moderate level, employee performance will peak. However, this conclusion is derived from theoretical analyses and is not supported by empirical data. Finally, another view suggests that no relationship exists between them (Tănăsescu and Ramona-Diana, 2019). Indubitably, it presupposes that employees are rational beings (Lebesby and Benders, 2020). Per this view, work stress cannot motivate employees or influence their psychology and thus cannot impact their performance.

To further explain the aforementioned diverse views, positive psychology proposes that work stress includes two main categories: challenge stress and hindrance stress (Cavanaugh et al., 2000; LePine et al., 2005). Based on their views, challenge stress represents stress that positively affects employees' work attitudes and behaviors, which improves employee performance by increasing work responsibility; by contrast, hindrance stress negatively affects employees' work attitudes and behaviors, which reduces employee performance by increasing role ambiguity (Hon and Chan, 2013; Deng et al., 2019).

During the COVID-19 pandemic, SMEs have faced a relatively higher risk of salary reductions, layoffs, or corporate bankruptcy (Adam and Alarifi, 2021). Hence, the competition among enterprises has intensified; managers may transfer some stress to employees, who, in turn, need to bear this to maintain and seek current and future career prospects, respectively (Lai et al., 2015). In this context, employee work stress stems from increased survival problems of SMEs, and such an external shock precipitates greater stress among employees than ever before (Gao, 2021). Stress more frequently manifests as hindrance stress (LePine et al., 2004), which negatively affects employees' wellbeing and quality of life (Orfei et al., 2022). It imposes a burden on employees, who need to spend time and energy coping with the stress. From the perspective of stressors, SMEs have faced serious survival problems during the COVID-19 pandemic, and consequently, employees have faced greater hindrance stress, thereby decreasing their performance. Hence, we propose the following hypothesis:

**H1**. Work stress negatively influences employee performance in SMEs during the COVID-19 pandemic.

### Work stress and mental health

According to the demand–control–support (DCS) model (Karasek and Theorell, 1990), high-stress work—such as high job demands, low job control, and low social support at work—may trigger health problems in employees over time (e.g., mental health problems; Chou et al., 2015; Park et al., 2016; Lu et al., 2020). The DCS model considers stress as an individual's response to perceiving high-intensity work (Houtman et al., 2007), which precipitates a change in the employee's cognitive, physical, mental, and emotional status. Of these, mental health problems including irritability, nervousness, aggressive behavior, inattention, sleep, and memory disturbances are a typical response to work stress (Mayerl et al., 2016; Neupane and Nygard, 2017). If the response persists for a considerable period, mental health problems such as anxiety or depression may occur (Bhui et al., 2012; Eskilsson et al., 2017). As coping with work stress requires an employee to exert continuous effort and apply relevant skills, it may be closely related to certain psychological problems (Poms et al., 2016; Harrison and Stephens, 2019).

The COVID-19 pandemic has disrupted the normal operating order of enterprises as well as employees' work rhythm. Consequently, employees might have faced greater challenges during this period (Piccarozzi et al., 2021). In this context, work stress includes stress related to health and safety risk, impaired performance, work adjustment, and negative emotions, for instance, such work stress can lead to unhealthy mental problems. Hence, we propose the following hypothesis:

**H2**. Work stress negatively influences mental health in SMEs during the COVID-19 pandemic.

### Mediating role of mental health

Previous research has found that employees' mental health status significantly affects their performance (Bubonya et al., 2017; Cohen et al., 2019; Soeker et al., 2019), the main reasons of which are as follows: First, mental health problems reduce employees' focus on their work, which is potentially detrimental to their performance (Hennekam et al., 2020). Second, mental health problems may render employees unable to work (Heffernan and Pilkington, 2011), which indirectly reduces work efficiency owing to increased sick leaves (Levinson et al., 2010). Finally, in the stress context, employees need to exert additional effort to adapt to the environment, which, consequently, make them feel emotionally exhausted. Hence, as their demands remain unfulfilled, their work satisfaction and performance decrease (Khamisa et al., 2016).

Hence, we propose that work stress negatively impacts mental health, which, in turn, positively affects employee performance. In other words, we argue that mental health mediates the relationship between work stress and employee performance. During the COVID-19 pandemic, work stress—owing to changes in the external environment—might have caused nervous and anxious psychological states in employees (Tan et al., 2020). Consequently, it might have rendered employees unable to devote their full attention to their work, and hence, their work performance might have decreased. Meanwhile, due to the pandemic, employees have faced the challenges of unclear job prospects and reduced income. Therefore, mental health problems manifest as moods characterized by depression and worry (Karatepe et al., 2020). Negative emotions negatively impact employee performance. Per the aforementioned arguments and hypothesis 2, we propose the following hypothesis:

**H3**. Mental health mediates the relationship between work stress and employee performance in SMEs during the COVID-19 pandemic.

### Moderating role of servant leadership

According to the upper echelons theory, leaders significantly influence organizational activities, and their leadership behavior influences the thinking and understanding of tasks among employees in enterprises (Hambrick and Mason, 1984). Servant leadership is a typical leadership behavior that refers to leaders exhibiting humility, lending power to employees, raising the moral level of subordinates, and placing the interests of employees above their own (Sendjaya, 2015; Eva et al., 2019). This leadership behavior provides emotional support to employees and increase their personal confidence and self-esteem and thus reduce negative effects of work stress. In our study, we propose that servant leadership reduces the negative effects of work stress on mental health in SMEs.

Servant leadership can reduce negative effects of work stress on mental health in the following ways: Servant leaders exhibit empathy and compassion (Lu et al., 2019), which help alleviate employees' emotional pain caused by work stress. Song et al. (2020) highlighted that work stress can cause psychological pain among employees. However, servant leaders are willing to listen to their employees and become acquainted with them, which facilitates communication between the leader and the employee (Spears, 2010). Hence, servant leadership may reduce employees' psychological pain through effective communication. Finally, servant leaders lend employees power, which makes the employees feel trusted. Employees—owing to their trust in the leaders—trust the enterprises as well, which reduces the insecurity caused by work stress (Phong et al., 2018). In conclusion, servant leadership serves as a coping resource that reduces the impact of losing social support and thus curbs negative employee emotions (Ahmed et al., 2021). Based on the aforementioned analysis, we find that servant leaders can reduce the mental health problems caused by work stress. Hence, we propose the following hypothesis:

**H4**. Servant leadership reduces the negative relationship between work stress and mental health in SMEs during the COVID-19 pandemic.

## Methodology

### Data collection and samples

To assess our theoretical hypotheses, we collected data by administering a questionnaire survey. The questionnaire was administered anonymously, and the respondents were informed regarding the purpose of the study. Owing to the impact of the pandemic, we distributed and collected the questionnaires by email. Specifically, we utilized the network relationships of our research group with the corporate campus and group members to distribute the questionnaires. In addition, to ensure the quality of the questionnaires, typically senior employees who had worked for at least 2 years at their enterprises were chosen as the respondents.

Before the formal survey, we conducted a pilot test. Thereafter, we revised the questionnaire based on the results of the trial investigation. Subsequently, we randomly administered the questionnaires to the target enterprises. Hence, 450 questionnaires were administered via email, and 196 valid questionnaires were returned—an effective rate of 43.6%. Table 1 presents the profiles of the samples.

**Table 1:** Profiles of the samples.

**Characteristics**		***N***	**Percent (%)**
Firm size (number of employees)	1–20	18	9.2
	21–50	80	40.8
	51–200	76	38.8
	201–500	22	11.2
Industry	High technology	71	36.2
	Non-high technology	125	63.8
Work age	3 years or less	63	32.1
	3–10 years	64	32.7
	More than 10 years	69	35.2

Table 1 shows the descriptive statistics of the sample. Based on the firm size, respondents who worked in a company with 1–20 employees accounted for 9.2%, those in a company with 21–50 employees accounted for 40.8%, those in a company with 51–200 employees accounted for 38.8%, and those in a company with 201–500 employees accounted for 11.2%. Regarding industry, the majority of the respondents (63.8%) worked for non-high-technology industry and 36.2% of the respondents worked for high-technology industry. Regarding work age, the participants with a work experience of 3 years or less accounted for 32.1%, those with work experience of 3–10 years accounted for 32.7%, and those with a work experience of more than 10 years accounted for 35.2%.

### Measures

Core variables in this study include English-version measures that have been well tested in prior studies; some modifications were implemented during the translation process. As the objective of our study is SMEs in China, we translated the English version to Chinese; this translation was carried out by two professionals to ensure accuracy. Thereafter, we administered the questionnaires to the respondents. Hence, as the measures of our variables were revised based on the trial investigation, we asked two professionals to translate the Chinese version of the responses to English to enable publishing this work in English. We evaluated all the items pertaining to the main variables using a seven-point Likert scale (7 = very high/strongly agree, 1 = very low/strongly disagree). The variable measures are presented subsequently.

#### Work stress (WS)

Following the studies of Parker and DeCotiis (1983) and Shah et al. (2021), we used 12 items to measure work stress, such as “I get irritated or nervous because of work” and “Work takes a lot of my energy, but the reward is less than the effort.”

#### Mental health (MH)

The GHQ-12 is a widely used tool developed to assess the mental health status (Liu et al., 2022). However, we revised the questionnaire by combining the research needs and results of the pilot test. We used seven items to measure mental health, such as “I feel that I am unable (or completely unable) to overcome difficulties in my work or life.” In the final calculation, the scoring questions for mental health were converted; higher scores indicated higher levels of mental health.

#### Servant leadership (SL)

Following the studies by Ehrhart (2004) and Sendjaya et al. (2019), we used nine items to measure servant leadership, including “My leader makes time to build good relationships with employees” and “My leader is willing to listen to subordinates during decision-making.”

#### Employee performance (EP)

We draw on the measurement method provided by Chen et al. (2002) and Khorakian and Sharifirad (2019); we used four items to represent employee performance. An example item is as follows: “I can make a contribution to the overall performance of our enterprise.”

#### Control variables

We controlled several variables that may influence employee performance, including firm size, industry, and work age. Firm size was measured by the number of employees. For industry, we coded them into two dummy variables (high-technology industry = 1, non-high-technology industry = 0). We calculated work experience by the number of years the employee has worked for the enterprise.

### Common method bias

Common method bias may exist because each questionnaire was completed independently by each respondent (Cai et al., 2017). We conducted a Harman one-factor test to examine whether common method bias significantly affected our data (Podsakoff and Organ, 1986); the results revealed that the largest factor in our data accounted for only 36.219% of the entire variance. Hence, common method bias did not significantly affect on our study findings.

### Reliability and validity

We analyzed the reliability and validity of our data for further data processing, the results of which are presented in Table 2. Based on these results, we found that Cronbach's alpha coefficient of each variable was >0.8, thus meeting the requirements for reliability of the variables. To assess the validity of each construct, we conducted four separate confirmatory factor analyses. All the factor loadings exceeded 0.5. Overall, the reliability and validity results met the requirements for further data processing.

**Table 2:** Results of confirmatory factor analysis and Cronbach's alpha coefficients.

**Variables**	**Items**	**Factor loading**	**Cronbach' α**
WS	WS1	0.655	0.910
	WS2	0.695	
	WS3	0.794	
	WS4	0.765	
	WS5	0.834	
	WS6	0.770	
	WS7	0.691	
	WS8	0.587	
	WS9	0.738	
	WS10	0.670	
	WS11	0.697	
	WS12	0.609	
MH	MH1	0.714	0.914
	MH2	0.849	
	MH3	0.762	
	MH4	0.856	
	MH5	0.822	
	MH6	0.872	
	MH7	0.806	
EP	EP1	0.738	0.832
	EP2	0.777	
	EP3	0.865	
	EP4	0.887	
SL	SL1	0.882	0.961
	SL2	0.877	
	SL3	0.879	
	SL4	0.868	
	SL5	0.898	
	SL6	0.832	
	SL7	0.875	
	SL8	0.856	
	SL9	0.906	

## Results

To verify our hypotheses, we used a hierarchical linear regression method. Before conducting the regression analysis, we performed a Pearson correlation analysis, the results of which are presented in Table 3.

**Table 3:** Descriptive statistics and correlation analysis.

**Variable**	**1**	**2**	**3**	**4**	**5**	**6**	**7**
1.Firm size	1						
2.Industry	−0.105	1					
3.Work age	0.102	−0.073	1				
4.WS	0.189**	0.045	−0.074	1			
5.MH	−0.110	−0.024	0.053	−0.494**	1		
6.SL	−0.123	−0.018	0.061	−0.273**	0.449**	1	
7.EP	−0.016	0.033	0.083	−0.228**	0.440**	0.556**	1
Mean	2.52	0.64	5.11	3.98	4.72	5.22	5.15
S. D.	0.813	0.482	2.461	1.197	1.266	1.125	1.005

^*^p < 0.05,

^**^p < 0.01,

^***^p < 0.001.

In the regression analysis, we calculated the variance inflation factor (VIF) of each variable and found that the VIF value of each variable was <3. Hence, the effect of multiple co-linearity is not significant. The results of regression analysis are presented in Tables 4, 5.

**Table 4:** Results of linear regression analysis (models 1–6).

**Variable**	**EP**				**MH**	
	**Model 1**	**Model 2**	**Model 3**	**Model 4**	**Model 5**	**Model 6**
Firm size	−0.027	0.032	0.039	0.043	−0.187	−0.032
Industry	0.077	0.106	0.107	0.109	−0.085	−0.008
Work age	0.036	0.028	0.025	0.024	0.032	0.010
WS		−0.193**		−0.016		−0.517***
MH			0.350***	0.343***		
*F*-value	0.575	3.013*	11.950***	9.526***	1.131	15.459***
*R* ^2^	0.009	0.059	0.200	0.200	0.017	0.245
Adj-*R*^2^	−0.007	0.040	0.183	0.179	0.002	0.229

^*^p < 0.05,

^**^p < 0.01,

^***^p < 0.001.

**Table 5:** Results of linear regression analysis (models 7–9).

**Variable**	**MH**		
	**Model 7**	**Model 8**	**Model 9**
Firm size	−0.187	0.012	0.011
Industry	−0.085	0.002	0.001
Work age	0.032	0.001	0.002
WS		−0.426***	−0.434***
SL		0.383***	0.387***
WS × SL			0.030
F value	1.131	20.548***	17.134***
*R* ^2^	0.017	0.351	0.352
Adj-R^2^	0.002	0.334	0.332

^*^p < 0.05,

^**^p < 0.01,

^***^p < 0.001.

Table 4 shows that model 1 is the basic model assessing the effects of control variables on employee performance. In model 2, we added an independent variable (work stress) to examine its effect on employee performance. The results revealed that work stress negatively affects employee performance (β = −0.193, *p* < 0.01). Therefore, hypothesis 1 is supported. Model 5 is the basic model that examines the effects of control variables on mental health. In model 6, we added an independent variable (work stress) to assess its effect on mental health. We found that work stress negatively affects mental health (β = −0.517, *p* < 0.001). Therefore, hypothesis 2 is supported.

To verify the mediating effect of mental health on the relationship between work stress and employee performance, we used the method introduced by Kenny et al. (1998), which is described as follows: (1) The independent variable is significantly related to the dependent variable. (2) The independent variable is significantly related to the mediating variable. (3) The mediating variable is significantly related to the dependent variable after controlling for the independent variable. (4) If the effect of the independent variable on the dependent variable becomes smaller, it indicates a partial mediating effect. (5) If the effect of the independent variable on the dependent variable is no longer significant, it indicates a full mediating effect. Based on this method, in model 4, mental health is significantly positively related to employee performance (β = 0.343, *p* < 0.001), and no significant correlation exists between work stress and employee performance (β = −0.016, *p* > 0.05). Hence, mental health fully mediates the relationship between work stress and employee performance. Therefore, hypothesis 3 is supported.

To verify the moderating effect of servant leadership on the relationship between work stress and mental health, we gradually added independent variables, a moderator variable, and interaction between the independent variables and moderator variable to the analysis, the results of which are presented in Table 5. In model 9, the moderating effect of servant leadership is not supported (β = 0.030, *p* > 0.05). Therefore, hypothesis 4 is not supported.

## Discussion

For SMEs, employees are core assets and crucial to their survival and growth (Shan et al., 2022). Specifically, owing to the COVID-19 pandemic, employees' work stress may precipitate burnout (Choi et al., 2019; Barello et al., 2020), which influences their performance. Researchers and practitioners have significantly focused on resolving the challenge of work stress (Karatepe et al., 2020; Tan et al., 2020; Gao, 2021). However, previous research has not clearly elucidated the relationship among work stress, mental health, servant leadership, and employee performance. Through this study, we found the following results:

Employees in SMEs face work stress owing to the COVID-19 pandemic, which reduces their performance. Facing these external shocks, survival and growth of SMEs may become increasingly uncertain (Adam and Alarifi, 2021). Employees' career prospects are negatively impacted. Meanwhile, the pandemic has precipitated a change in the way employees work, their workspace, and work timings. Moreover, their work is now intertwined with family life. Hence, employees experience greater stress at work than ever before (Gao, 2021), which, in turn, affects their productivity and deteriorates their performance.

Furthermore, we found that mental health plays a mediating role in the relationship between work stress and employee performance; this suggests that employees' mental status is influenced by work stress, which, in turn, lowers job performance. Per our findings, due to the COVID-19 pandemic, employees experience nervous and anxious psychological states (Tan et al., 2020), which renders them unable to devote their full attention to their work; hence, their work performance is likely to decrease.

Finally, we found that leaders are the core of any enterprise (Ahn et al., 2018). Hence, their leadership behavior significantly influences employees. Per previous research, servant leadership is considered a typical leadership behavior characterized by exhibiting humility, delegating power to employees, raising the morale of subordinates, and placing the interests of employees above their own (Sendjaya, 2015; Eva et al., 2019). Through theoretical analysis, we found that servant leadership mitigates the negative effect of work stress on mental health. However, the empirical results are not significant possibly because work stress of employees in SMEs is rooted in worries regarding the future of the macroeconomic environment, and the resulting mental health problems cannot be cured merely by a leader.

Hence, due to the COVID-19 pandemic, employees experience work stress, which precipitates mental health problems and poor employee performance. To solve the problem of work stress, SMEs should pay more attention to fostering servant leadership. Meanwhile, organizational culture is also important in alleviating employees' mental health problems and thus reducing negative effects of work stress on employee performance.

## Implications

This study findings have several theoretical and managerial implications.

### Theoretical implications

First, per previous research, no consistent conclusion exists regarding the relationship between work stress and employee performance, including positive relationships (Ismail et al., 2015; Soomro et al., 2019), negative relationships (Yunus et al., 2018; Nawaz Kalyar et al., 2019; Purnomo et al., 2021), inverted U-shaped relationships (McClenahan et al., 2007; Hamidi and Eivazi, 2010), and no relationship (Tănăsescu and Ramona-Diana, 2019). We report that work stress negatively affects employee performance in SMEs during the COVID-19 pandemic; thus, this study contributes to the understanding of the situational nature of work stress and provides enriching insights pertaining to positive psychology.

Second, we established the research path that work stress affects employee performance. Mental health is a psychological state that may influence an individual's work efficiency. In this study, we explored its mediating role, which opens the black box of the relationship between work stress and employee performance; thus, this study contributes to a greater understanding of the role of work stress during the COVID-19 pandemic.

Finally, this study sheds light on the moderating effect of servant leadership, which is useful for understanding why some SMEs exhibit greater difficulty in achieving success than others during the COVID-19 pandemic. Previous research has explained the negative effect of work stress (Yunus et al., 2018; Nawaz Kalyar et al., 2019; Purnomo et al., 2021). However, few studies have focused on how to resolve the problem. We identify servant leadership as the moderating factor providing theoretical support for solving the problem of work stress. This study expands the explanatory scope of the upper echelons theory.

### Practice implications

First, this study elucidates the sources and mechanisms of work stress in SMEs during the COVID-19 pandemic. Employees should continuously acquire new skills to improve themselves and thus reduce their replaceability. Meanwhile, they should enhance their time management and emotional regulation skills to prevent the emergence of adverse psychological problems.

Second, leaders in SMEs should pay more attention to employees' mental health to prevent the emergence of hindrance stress. Employees are primarily exposed to stress from health and safety risks, impaired performance, and negative emotions. Hence, leaders should communicate with employees in a timely manner to understand their true needs, which can help avoid mental health problems due to work stress among employees.

Third, policymakers should realize that a key cause of employee work stress in SMEs is attributable to concerns regarding the macroeconomic environment. Hence, they should formulate reasonable support policies to improve the confidence of the whole society in SMEs, which helps mitigate SME employees' work stress during emergency events like the COVID-19 pandemic.

Finally, as work stress causes mental health problems, SME owners should focus on their employees' physical as well as mental health. Society should establish a psychological construction platform for SME employees to help them address their psychological problems.

## Limitations and future research

This study has limitations, which should be addressed by further research. First, differences exist in the impact of the pandemic on different industries. Future research should focus on the impact of work stress on employee performance in different industries. Second, this study only explored the moderating role of servant leadership. Other leadership behaviors of leaders may also affect work stress. Future research can use case study methods to explore the role of other leadership behaviors.

## Conclusion

This study explored the relationship between work stress and employee performance in SMEs during the COVID-19 pandemic. Using a sample of 196 SMEs from China, we found that as a typical result of emergency events, work stress negatively affects employees' performance, particularly by affecting employees' mental health. Furthermore, we found that servant leadership provides a friendly internal environment to mitigate negative effects of work stress on employees working in SMEs.

## Data availability statement

The original contributions presented in the study are included in the article/supplementary material, further inquiries can be directed to the corresponding author/s.

## Ethics statement

Ethical review and approval was not required for the study on human participants in accordance with the local legislation and institutional requirements. Written informed consent from the patients/participants or patients/participants legal guardian/next of kin was not required to participate in this study in accordance with the national legislation and the institutional requirements.

## Author contributions

BC: conceptualization, methodology, writing—original draft, and visualization. LW: formal analysis. BL: investigation, funding acquisition, and writing—review and editing. WL: resources, project administration, and supervision. All authors contributed to the article and approved the submitted version.

## Funding

This research was supported by the major project of Henan Province Key R&D and Promotion Special Project (Soft Science) Current Situation, Realization Path and Guarantee Measures for Digital Transformation Development of SMEs in Henan Province under the New Development Pattern (Grant No. 222400410159).

## Conflict of interest

The authors declare that the research was conducted in the absence of any commercial or financial relationships that could be construed as a potential conflict of interest.

## Publisher's note

All claims expressed in this article are solely those of the authors and do not necessarily represent those of their affiliated organizations, or those of the publisher, the editors and the reviewers. Any product that may be evaluated in this article, or claim that may be made by its manufacturer, is not guaranteed or endorsed by the publisher.

## References

[B1] Adam N. A. Alarifi G. (2021). Innovation practices for survival of small and medium enterprises (SMEs) in the COVID-19 times: the role of external support. J. Innov. Entrepreneursh. 10, 1–22. 10.1186/s13731-021-00156-6 PMC8155652 34075328

[B2] Ahmed I. Ali M. Usman M. Syed K. H. Rashid H. A. (2021). Customer Mistreatment and Insomnia in Employees-a Study in Context of COVID-19. J. Behav. Sci. 31, 248–271.

[B3] Ahn J. Lee S. Yun S. (2018). Leaders' core self-evaluation, ethical leadership, and employees' job performance: the moderating role of employees' exchange ideology. J. Bus. Ethics. 148, 457–470. 10.1007/s10551-016-3030-0

[B4] Barello S. Palamenghi L. Graffigna G. (2020). Burnout and somatic symptoms among frontline healthcare professionals at the peak of the Italian COVID-19 pandemic. Psychiatry Res. 290, 113129. 10.1016/j.psychres.2020.113129 32485487 PMC7255285

[B5] Bhui K. S. Dinos S. Stansfeld S. A. White P. D. (2012). A synthesis of the evidence for managing stress at work: a review of the reviews reporting on anxiety, depression, and absenteeism. J. Environ. Public Health. 2012. 10.1155/2012/515874 22496705 PMC3306941

[B6] Bubonya M. Cobb-Clark D. A. Wooden M. (2017). Mental health and productivity at work: Does what you do matter?. Labour Econ. 46, 150–165. 10.1016/j.labeco.2017.05.001

[B7] Cai L. Chen B. Chen J. Bruton G. D. (2017). Dysfunctional competition and innovation strategy of new ventures as they mature. J. Bus. Res. 78, 111–118. 10.1016/j.jbusres.2017.05.008

[B8] Cavanaugh M. A. Boswell W. R. Roehling M. V. Boudreau J. W. (2000). An empirical examination of self-reported work stress among US managers. J. Appl. Psychol. 85, 65–74. 10.1037/0021-9010.85.1.65 10740957

[B9] Chen I. S. Fellenz M. R. (2020). Personal resources and personal demands for work engagement: Evidence from employees in the service industry. Int. J. Hosp. Manage. 90, 102600. 10.1016/j.ijhm.2020.102600 32834349 PMC7326455

[B10] Chen Z. X. Tsui A. S. Farh J. L. (2002). Loyalty to supervisor vs. organizational commitment: Relationships to employee performance in China. J. Occupa. Organ. Psychol. 75, 339–356. 10.1348/096317902320369749

[B11] Choi H. M. Mohammad A. A. Kim W. G. (2019). Understanding hotel frontline employees' emotional intelligence, emotional labor, job stress, coping strategies and burnout. Int. J. Hosp. Manage. 82, 199–208. 10.1016/j.ijhm.2019.05.002

[B12] Chou L. Hu S. Lo M. (2015). Job stress, mental health, burnout and arterial stiffness: a cross-sectional study among Taiwanese medical professionals. Atherosclerosis. 241, e135–e135. 10.1016/j.atherosclerosis.2015.04.468

[B13] Cohen J. Schiffler F. Rohmer O. Louvet E. Mollaret P. (2019). Is disability really an obstacle to success? Impact of a disability simulation on motivation and performance. J. Appl. Soc. Psychol. 49, 50–59. 10.1111/jasp.12564

[B14] Deng J. Guo Y. Ma T. Yang T. Tian X. (2019). How job stress influences job performance among Chinese healthcare workers: a cross-sectional study. Environ. Health Prevent. Med. 24, 1–11. 10.1186/s12199-018-0758-4 30611191 PMC6320635

[B15] Eggers F. (2020). Masters of disasters? Challenges and opportunities for SMEs in times of crisis. J. Bus. Res. 116, 199–208. 10.1016/j.jbusres.2020.05.025 32501306 PMC7247971

[B16] Ehrhart M. G. (2004). Leadership and procedural justice climate as antecedents of unit-level organizational citizenship behavior. Pers. Psychol. 57, 61–94. 10.1111/j.1744-6570.2004.tb02484.x

[B17] Eskilsson T. Slunga Järvholm L. Malmberg Gavelin H. Stigsdotter Neely A. Boraxbekk C. J. (2017). Aerobic training for improved memory in patients with stress-related exhaustion: a randomized controlled trial. BMC psychiatry. 17, 1–10. 10.1186/s12888-017-1457-1 28865430 PMC5581420

[B18] Eva N. Robin M. Sendjaya S. Van Dierendonck D. Liden R. C. (2019). Servant leadership: a systematic review and call for future research. Leadersh. Q. 30, 111–132. 10.1016/j.leaqua.2018.07.004

[B19] Flynn A. (2017). Re-thinking SME disadvantage in public procurement. J. Small Bus. Enter. Dev. 24, 991–1008. 10.1108/JSBED-03-2017-0114

[B20] Gao Y. (2021). Challenges and countermeasures of human resource manage in the post-epidemic Period. Int. J. Manage Educ. Human Dev. 1, 036–040.

[B21] Green N. Tappin D. Bentley T. (2020). Working from home before, during and after the Covid-19 pandemic: implications for workers and organisations. N. Z. J. Employ. Relat. 45, 5–16. 10.24135/nzjer.v45i2.1935514111

[B22] Hambrick D. C. Mason P. A. (1984). Upper echelons: the organization as a reflection of its top managers. Acad.Manage. Rev. 9, 193–206. 10.2307/258434

[B23] Hamidi Y. Eivazi Z. (2010). The relationships among employees' job stress, job satisfaction, and the organizational performance of Hamadan urban health centers. Soc. Behav. Pers. Int. J. 38, 963–968. 10.2224/sbp.2010.38.7.963

[B24] Harrison M. A. Stephens K. K. (2019). Shifting from wellness at work to wellness in work: Interrogating the link between stress and organization while theorizing a move toward wellness-in-practice. Manage. Commun. Q. 33, 616–649. 10.1177/0893318919862490

[B25] Haslam S. A. Reicher S. D. Platow M. J. (2020). The New Psychology of Leadership: Identity, Influence and Power. London, UK: Routledge. 10.4324/9781351108232

[B26] Heffernan J. Pilkington P. (2011). Supported employment for persons with mental illness: systematic review of the effectiveness of individual placement and support in the UK. J. Mental Health. 20, 368–380. 10.3109/09638237.2011.556159 21332324

[B27] Hennekam S. Richard S. Grima F. (2020). Coping with mental health conditions at work and its impact on self-perceived job performance. Employee Relat. Int. J. 42, 626–645. 10.1108/ER-05-2019-021135250327

[B28] Hon A. H. Chan W. W. (2013). The effects of group conflict and work stress on employee performance. Cornell Hosp. Q. 54, 174–184. 10.1177/1938965513476367

[B29] Houtman I. Jettinghof K. Cedillo L. (2007). Raising Awareness of Stress at Work in Developing Countires: a Modern Hazard in a Traditional Working Environment: Advice to Employers and Worker Representatives. Geneva: World Health Organization.

[B30] Ismail A. Saudin N. Ismail Y. Samah A. J. A. Bakar R. A. Aminudin N. N.. (2015). Effect of workplace stress on job performance. Econ. Rev. J. Econ. Bus. 13, 45–57.

[B31] Karasek R. Theorell T. (1990). Healthy Work: Stress, Productivity, and the Reconstruction of Working Life. New York: Basic Books.

[B32] Karatepe O. M. Rezapouraghdam H. Hassannia R. (2020). Job insecurity, work engagement and their effects on hotel employees' non-green and nonattendance behaviors. Int. J. Hosp. Manage. 87, 102472. 10.1016/j.ijhm.2020.102472

[B33] Kenny D. Kashy D. Bolger N. (1998). Data analysis in social psychology. Handbook Soc. Psychol. 1, 233–268.

[B34] Khamisa N. Peltzer K. Ilic D. Oldenburg B. (2016). Work related stress, burnout, job satisfaction and general health of nurses: a follow-up study. Int. J. Nurs. Pract. 22, 538–545. 10.1111/ijn.12455 27241867

[B35] Khorakian A. Sharifirad M. S. (2019). Integrating implicit leadership theories, leader–member exchange, self-efficacy, and attachment theory to predict job performance. Psychol. Rep. 122, 1117–1144. 10.1177/0033294118773400 29764300

[B36] Lai H. Hossin M. A. Li J. Wang R. Hosain M. S. (2022). Examining the relationship between COVID-19 related job stress and employees' turnover intention with the moderating role of perceived organizational support: Evidence from SMEs in China. Int. J. Environ. Res. Public Health. 19, 3719. 10.3390/ijerph19063719 35329404 PMC8953488

[B37] Lai Y. Saridakis G. Blackburn R. (2015). Job stress in the United Kingdom: Are small and medium-sized enterprises and large enterprises different?. Stress Health. 31, 222–235. 10.1002/smi.2549 24302431

[B38] Lebesby K. Benders J. (2020). Too smart to participate? Rational reasons for employees' non-participation in action research. Syst. Pract. Action Res. 33, 625–638. 10.1007/s11213-020-09538-5

[B39] LePine J. A. LePine M. A. Jackson C. L. (2004). Challenge and hindrance stress: relationships with exhaustion, motivation to learn, and learning performance. J. Appl. Psychol. 89, 883. 10.1037/0021-9010.89.5.883 15506867

[B40] LePine J. A. Podsakoff N. P. LePine M. A. (2005). A meta-analytic test of the challenge stressor–hindrance stressor framework: an explanation for inconsistent relationships among stressors and performance. Acad. Manage J. 48, 764–775. 10.5465/amj.2005.18803921

[B41] Levinson D. Lakoma M. D. Petukhova M. Schoenbaum M. Zaslavsky A. M. Angermeyer M.. (2010). Associations of serious mental illness with earnings: results from the WHO World Mental Health surveys. Br. J. Psychiatry. 197, 114–121. 10.1192/bjp.bp.109.073635 20679263 PMC2913273

[B42] Li S. Rees C. J. Branine M. (2019). Employees' perceptions of human resource Manage practices and employee outcomes: empirical evidence from small and medium-sized enterprises in China. Employee Relat. Int. J. 41, 1419–1433. 10.1108/ER-01-2019-0065

[B43] Liu Z. Xi C. Zhong M. Peng W. Liu Q. Chu J.. (2022). Factorial validity of the 12-item general health questionnaire in patients with psychological disorders. Current Psychol. 1–9. 10.1007/s12144-022-02845-1

[B44] Lu A. C. C. Gursoy D. (2016). Impact of job burnout on satisfaction and turnover intention: do generational differences matter?. J. Hosp. Tour. Res. 40, 210–235. 10.1177/1096348013495696

[B45] Lu J. Zhang Z. Jia M. (2019). Does servant leadership affect employees' emotional labor? A Soc. information-processing perspective. J. Bus. Ethics. 159, 507–518. 10.1007/s10551-018-3816-3

[B46] Lu L. (1997). Hassles, appraisals, coping and distress: a closer look at the stress process. Kaohsiung J. Med. Sci. 13, 503–510. 9311202

[B47] Lu Y. Zhang Z. Yan H. Rui B. Liu J. (2020). Effects of occupational hazards on job stress and mental health of factory workers and miners: a propensity score analysis. BioMed Res. Int. 2020. 10.1155/2020/1754897 32904478 PMC7456464

[B48] Mansour S. Tremblay D. G. (2018). Work–family conflict/family–work conflict, job stress, burnout and intention to leave in the hotel industry in Quebec (Canada): moderating role of need for family friendly practices as “resource passageways”. Int. J. Human Res. Manage. 29, 2399–2430. 10.1080/09585192.2016.1239216

[B49] Mayerl H. Stolz E. Waxenegger A. Rásky É. Freidl W. (2016). The role of personal and job resources in the relationship between psychoSoc. job demands, mental strain, and health problems. Front. Psychol. 7, 1214. 10.3389/fpsyg.2016.01214 27582717 PMC4987342

[B50] McClenahan C. Shevlin M. G. Bennett C. O'Neill B. (2007). Testicular self-examination: a test of the health belief model and the theory of planned behaviour. Health Educ. Res. 22, 272–284. 10.1093/her/cyl076 16885203

[B51] Nawaz Kalyar M. Shafique I. Ahmad B. (2019). Job stress and performance nexus in tourism industry: a moderation analysis. Tour. Int. Interdiscip. J. 67, 6–21.

[B52] Neupane S. Nygard C. H. (2017). Physical and mental strain at work: relationships with onset and persistent of multi-site pain in a four-year follow up. Int. J. Indus. Ergon. 60, 47–52. 10.1016/j.ergon.2016.03.005

[B53] Olugbade O. A. Karatepe O. M. (2019). Stressors, work engagement and their effects on hotel employee outcomes. Serv. Indus. J. 39, 279–298. 10.1080/02642069.2018.1520842

[B54] Orfei M. D. Porcari D. E. D'Arcangelo S. Maggi F. Russignaga D. Lattanzi N.. (2022). COVID-19 and stressful adjustment to work: a long-term prospective study about homeworking for bank employees in Italy. Front. Psychol. 13, 843095. 10.3389/fpsyg.2022.843095 35369135 PMC8970302

[B55] Park S. K. Rhee M. K. Barak M. M. (2016). Job stress and mental health among nonregular workers in Korea: What dimensions of job stress are associated with mental health?. Arch. Environ. Occup. Health. 71, 111–118. 10.1080/19338244.2014.997381 25513849

[B56] Parker D. F. DeCotiis T. A. (1983). Organizational determinants of job stress. Organ. Behav. Human Perform. 32, 160–177. 10.1016/0030-5073(83)90145-9

[B57] Phong L. B. Hui L. Son T. T. (2018). How leadership and trust in leaders foster employees' behavior toward knowledge sharing. Soc. Behav. Pers. Int. J. 46, 705–720. 10.2224/sbp.6711

[B58] Piccarozzi M. Silvestri C. Morganti P. (2021). COVID-19 in manage studies: a systematic literature review. Sustainability. 13, 3791. 10.3390/su13073791

[B59] Podsakoff P. M. Organ D. W. (1986). Self-reports in organizational research: problems and prospects. J. Manage. 12, 531–544. 10.1177/0149206386012004088452065

[B60] Poms L. W. Fleming L. C. Jacobsen K. H. (2016). Work-family conflict, stress, and physical and mental health: a model for understanding barriers to and opportunities for women's well-being at home and in the workplace. World Med. Health Policy. 8, 444–457. 10.1002/wmh3.211

[B61] Prasad K. Vaidya R. W. (2020). Association among Covid-19 parameters, occupational stress and employee performance: an empirical study with reference to Agricultural Research Sector in Hyderabad Metro. Sustain. Humanosphere. 16, 235–253.

[B62] Purnomo K. S. H. Lustono L. Tatik Y. (2021). The effect of role conflict, role ambiguity and job stress on employee performance. Econ. Educ. Anal. J. 10, 532–542. 10.15294/eeaj.v10i3.5079311529953

[B63] Richardson K. M. Rothstein H. R. (2008). Effects of occupational stress manage intervention programs: a meta-analysis. J. Occup. Health Psychol. 13, 69–93. 10.1037/1076-8998.13.1.69 18211170

[B64] Robbins S. P. (2005). Essentials of Organizational Behavior (8th ed.). Upper Saddle River, NJ: Prentice Hall.

[B65] Saleem F. Malik M. I. Qureshi S. S. (2021). Work stress hampering employee performance during COVID-19: is safety culture needed? *Front*. Psychol. 12, 655839. 10.3389/fpsyg.2021.655839 34512434 PMC8426577

[B66] Sendjaya S. (2015). Personal and Organizational Excellence Through Servant Leadership Learning to Serve, Serving to Lead, Leading to Transform. Berlin, Germany: Springer.

[B67] Sendjaya S. Eva N. Butar Butar I. Robin M. Castles S. (2019). SLBS-6: Validation of a short form of the servant leadership behavior scale. J. Bus. Ethics. 156, 941–956. 10.1007/s10551-017-3594-3

[B68] Shah S. H. A. Haider A. Jindong J. Mumtaz A. Rafiq N. (2021). The impact of job stress and state anger on turnover intention among nurses during COVID-19: the mediating role of emotional exhaustion. Front. Psychol. 12. 10.3389/fpsyg.2021.810378 35222162 PMC8863937

[B69] Shan B. Liu X. Gu A. Zhao R. (2022). The effect of occupational health risk perception on job satisfaction. Int. J. Environ. Res. Public Health. 19, 2111. 10.3390/ijerph19042111 35206297 PMC8872356

[B70] Shimazu A. Nakata A. Nagata T. Arakawa Y. Kuroda S. Inamizu N.. (2020). PsychoSoc. impact of COVID-19 for general workers. J. Occup. Health. 62, e12132. 10.1002/1348-9585.12132 32515864 PMC7263354

[B71] Soeker M. S. Truter T. Van Wilgen N. Khumalo P. Smith H. Bezuidenhout S.. (2019). The experiences and perceptions of individuals diagnosed with schizophrenia regarding the challenges they experience to employment and coping strategies used in the open labor market in Cape Town, South Africa. Work. 62, 221–231. 10.3233/WOR-192857 30829633

[B72] Song L. Wang Y. Li Z. Yang Y. Li H. (2020). Mental health and work attitudes among people resuming work during the COVID-19 pandemic: a cross-sectional study in China. Int. J. Environ. Res. Public Health. 17, 5059. 10.3390/ijerph17145059 32674361 PMC7400483

[B73] Soomro M. A. Memon M. S. Bukhari N. S. (2019). Impact of stress on employees performance in public sector Universities of Sindh. Sukkur IBA J. Manage. Bus. 6, 114–129. 10.30537/sijmb.v6i2.327

[B74] Spears L. C. (2010). Character and servant leadership: ten characteristics of effective, caring leaders. J. Virtues Leadersh. 1, 25–30.

[B75] Tan W. Hao F. McIntyre R. S. Jiang L. Jiang X. Zhang L.. (2020). Is returning to work during the COVID-19 pandemic stressful? A study on immediate mental health status and psychoneuroimmunity prevention measures of Chinese workforce. Brain Behav. Immun. 87, 84–92. 10.1016/j.bbi.2020.04.055 32335200 PMC7179503

[B76] Tănăsescu R. I. Ramona-Diana L. E. O. N. (2019). Emotional intelligence, occupational stress and job performance in the Romanian banking system: a case study approach. Manage Dyn. Knowl. Econ. 7, 323–335. 10.25019/MDKE/7.3.03

[B77] Tiedtke C. Rijk D. E. Van den Broeck A. A. Godderis L. (2020). Employers' experience on involvement in sickness absence/return to work support for employees with Cancer in small enterprises. J. Occup. Rehabil. 30, 635–645. 10.1007/s10926-020-09887-x 32246294

[B78] Tu Y. Li D. Wang H. J. (2021). COVID-19-induced layoff, survivors' COVID-19-related stress and performance in hospital industry: the moderating role of social support. Int. J. Hosp. Manage. 95, 102912. 10.1016/j.ijhm.2021.102912 35702566 PMC9183452

[B79] Uchmanowicz I. Karniej P. Lisiak M. Chudiak A. Lomper K. Wiśnicka A.. (2020). The relationship between burnout, job satisfaction and the rationing of nursing care—A cross-sectional study. J. Nurs. Manage. 28, 2185–2195. 10.1111/jonm.13135 32805771

[B80] Xu S. Wang Y. C. Ma E. Wang R. (2020). Hotel employees' fun climate at work: effects on work-family conflict and employee deep acting through a collectivistic perspective. Int. J. Hosp. Manage. 91, 102666. 10.1016/j.ijhm.2020.102666 32934433 PMC7484734

[B81] Yu D. Yang K. Zhao X. Liu Y. Wang S. D'Agostino M. T.. (2022). Psychological contract breach and job performance of new generation of employees: Considering the mediating effect of job burnout and the moderating effect of past breach experience. *Front*. Psychol. 13, 985604. 10.3389/fpsyg.2022.985604 36160600 PMC9501888

[B82] Yunus N. H. Mansor N. Hassan C. N. Zainuddin A. Demong N. A. R. (2018). The role of supervisor in the relationship between job stress and job performance. Int. J. Acad. Res. Bus. Soc. Sci. 8, 1962–1970. 10.6007/IJARBSS/v8-i11/5560

